# Lepidium Meyenii Walp. (Maca) and Blood Biomarkers of Muscle Damage and Post-Exertion Protein Degradation: A Systematic Review and Meta-Analysis of Preclinical Studies

**DOI:** 10.3390/nu18122009

**Published:** 2026-06-20

**Authors:** Javiera Rodríguez Rojas, Álvaro Huerta Ojeda, Guillermo Barahona-Fuentes, Carlos Jorquera-Aguilera, Jorge Cancino-López, María-Mercedes Yeomans-Cabrera, Leonardo Pavez, Carlos Jara-Gutiérrez, Luis Javier Chirosa-Ríos

**Affiliations:** 1 Laboratorio de Fuerza y Acondicionamiento, Grupo de Investigación CTS-642, Departamento de Educación Física y Deportes, Universidad de Granada, 18071 Granada, Spain; e.jrodriguezr@go.ugr.es (J.R.R.); lchirosa@ugr.es (L.J.C.-R.); 2 Departamento de Ciencias Biomédicas, Facultad de Medicina, Universidad Católica del Norte, Coquimbo 1780000, Chile; 3 Núcleo de Investigación en Salud, Actividad Física y Deporte ISAFYD, Universidad de Las Américas, Viña del Mar 2531098, Chile; 4 Faculty of Education and Humanities, School of Sports Sciences, Universidad Andres Bello, Viña del Mar 2520000, Chile; guillermo.barahona@unab.cl; 5 Facultad de Ciencias, Escuela de Nutrición y Dietética, Universidad Mayor, Santiago 8580745, Chile; carlos.jorquera@mayor.cl; 6 Exercise Physiology and Metabolism Laboratory, Escuela de Kinesiología, Universidad Finis Terrae, Santiago 7501015, Chile; jcancino@uft.cl; 7 Escuela de Psicología, Facultad de Salud y Ciencias Sociales, Universidad de Las Américas, Viña del Mar 2531098, Chile; maria.yeomans@edu.udla.cl; 8 Núcleo de Investigación en Ciencias Biológicas (NICB), Facultad de Medicina Veterinaria y Agronomía, Universidad de Las Américas, Santiago 7500975, Chile; lpavez@udla.cl; 9 Departamento de Ciencias Químicas y Biológicas, Universidad Bernardo O’Higgins, Santiago 8370933, Chile; 10 Centro Interdisciplinario de Investigación Biomédica e Ingeniería para la Salud (MEDING), Escuela de Kinesiología, Facultad de Medicina, Universidad de Valparaíso, Valparaíso 2362735, Chile; carlos.jara@uv.cl

**Keywords:** *Lepidium meyenii*, maca, muscle damage post-exertion, creatine kinase, lactate dehydrogenase, blood urea nitrogen, animal model

## Abstract

**Background**: *Lepidium meyenii* Walp (*L. meyenii*), traditionally known as maca, is widely recognized for its health-promoting properties, including potential protection against exercise-induced muscle damage (EIMD). However, its precise effect on post-exercise blood biomarkers remains unclear. **Objective**: This study aimed to qualitatively review research published until April 2026 examining *L. meyenii* supplementation to reduce blood markers of muscle damage and protein degradation post-exertion in animal studies. Specifically, the effect size (ES) of *L. meyenii* supplementation on post-exercise levels of creatine kinase (CK), lactate dehydrogenase (LDH), and blood urea nitrogen (BUN) was estimated. **Methods**: This systematic review and meta-analysis were conducted in accordance with the PRISMA guidelines. The certainty of the evidence was assessed using the GRADE framework. Relevant studies were identified through Web of Science, Scopus, SPORTDiscus, PubMed, and MEDLINE. Eligible studies included in vivo experiments in animals with controlled designs and pre-/post-intervention assessments. Methodological quality and risk of bias were evaluated using the CAMARADES tool. Statistical analysis involved standardized mean differences (SMD) using Hedges’ g with 95% confidence intervals. **Results**: 15 studies were included in the systematic review, and 14 studies in animals in the meta-analysis. The CAMARADES scores ranged from 5 to 7 points, indicating moderate methodological quality. Supplementation with *L. meyenii* was not associated with statistically significant changes in LDH (SMD = −1.37; 95% CI −3.34 to 0.59), BUN (SMD = −0.37; 95% CI −2.16 to 1.42) nor CK (SMD = 0.29; 95% CI −5.45 to 6.03), with very high heterogeneity (I^2^ > 97%). Exploratory subgroup analyses and meta-regression analyses by formulation type and dose did not identify any moderators that could robustly explain this heterogeneity. **Conclusions**: The available evidence does not support a robust overall effect of *L. meyenii* supplementation on blood biomarkers of muscle damage or protein catabolism in animals subjected to physical stress. The high degree of heterogeneity could not be robustly explained by either the type of formulation or the dose. These findings, which are exploratory and hypothesis-generating in nature, highlight the need for standardized, well-characterized formulations and trials with adequate statistical power.

## 1. Introduction

Physical activity and structured training lead to significant improvements in overall physical fitness [1]. From a health perspective, maintaining an active lifestyle contributes to a better quality of life [2]. In addition, athletes must follow specialized training programs to get into shape [3]. In both cases, when these activities are performed excessively or without adequate control, they can trigger skeletal muscle disorders, including exercise-induced muscle damage (EIMD) [4]. To ensure that physical activity, physical training, or sports practice produce the expected effects without compromising the athlete’s health, they must be carefully regulated and supervised by professionals [5].

EIMD is a post-stress phenomenon of the neuromuscular system, characterized by transient morphological and functional alterations in both skeletal and nervous muscles [6]. These alterations can occur from the onset of physical activity up to 14 days after the exercise session that produces EIMD [7], with their peak intensity reaching between 12 and 48 h post-exertion [8]. This phenomenon is characterized by symptoms such as muscle pain, reduced contractile capacity, and structural alterations [9]; a reduction in the functional markers of muscle strength was also observed [10,11]. The morphological and physiological mechanisms by which high-intensity and/or strenuous exercise can produce muscle damage are varied [12]. Cellular analysis suggests that EIMD is caused by alterations in the extracellular matrix, degradation of structural proteins, and damage to myofibers during repeated sarcomere elongation [13,14].

The scientific literature indicates that the enzymes creatine kinase (CK) and lactate dehydrogenase (LDH) are reliable biomarkers for assessing EIMD [15]. In addition to the biomarkers described above, blood urea nitrogen (BUN) has been widely used as a marker of protein catabolism, hydration status, and overtraining [16].

Specifically, Macero et al. [17] investigated biomarker changes after EIMD in adolescent athletes, observing that CK is the primary marker of post-exertion muscle damage [17]. Additionally, Ozkan and Ibrahim [18] evaluated dehydration and EIMD in elite wrestlers, demonstrating a positive correlation between post-exertion CK and BUN levels [18]. These findings reinforce the importance of monitoring, through biomarkers, the impact of physical training programs and sports practice on athletes [19].

In addition to reflecting structural changes in the muscle and metabolic stress, EIMD is also accompanied by a complex inflammatory response that contributes to tissue repair and recovery [20]. Therefore, biomarkers of muscle damage and protein catabolism reflect physiological processes that occur in parallel with the inflammatory and recovery responses triggered by EIMD [21]. This response is characterized by an increase in the levels of proinflammatory markers such as C-reactive protein (CRP), interleukin 6 (IL-6), and tumor necrosis factor alpha (TNF-α) [21,22]. It has also been shown that these proinflammatory biomarkers increase in an intensity-dependent manner during physical exercise [23].

Despite these protective mechanisms, the presence of EIMD represents a problem for elite athletes and physically active individuals. Its consequences can range from muscle soreness to overtraining syndrome, as EIMD can reduce physical performance and significantly delay recovery [24]. For this reason, scientists and coaches strive to optimize physical training and sports practice safely, occasionally utilizing ergogenic nutritional aids such as supplements [25], thereby enabling optimal post-exertion recovery [26] and injury prevention [27].

One of these ergogenic aids is *L. meyenii*, also known as maca, a plant in the Brassicaceae family and native to the central Andes of Peru, where it grows at elevations between 3500 and 5000 m above sea level [28]. *L. meyenii*, due to its physiological properties, has also been considered a potential adaptogen. Preclinical studies have reported that supplementation with *L. meyenii* may alleviate post-exercise physiological stress. Specifically, some studies have observed a decrease in CK, LDH, and BUN levels following supplementation with different forms of *L. meyenii* in animals subjected to strenuous exercise [29,30,31]. These findings have sparked growing interest in the potential use of *L. meyenii* as a nutritional strategy to promote post-exercise recovery. Similar effects have been reported for other herbal supplements, such as *Panax ginseng* and *Rhodiola rosea* [32]. Evidence suggests that Panax ginseng, a root traditionally used in Asian medicine, may improve physical performance, particularly during activities performed at more than 70% of maximum oxygen consumption (VO_2_max) until exhaustion, both with acute and long-term supplementation [33]. Similarly, *Rhodiola rosea* has been linked to a delayed onset of fatigue during maximal incremental exercise and time-trial protocols. [34]. However, the bioavailability and profiles of these bioactive compounds vary considerably depending on the hypocotyl’s geographical origin, processing, and color [35]. It has been reported that *L. meyenii* grown in non-native regions, such as China, exhibits significant differences in phenotypic characteristics and bioactive compound content compared with *L. meyenii* grown in its place of origin, the Andean region of Peru [36]. Likewise, the processing method used for *L. meyenii* can significantly alter the quality and concentration of its bioactive compounds, including glucosinolates, macaenes, and macamides, thereby directly affecting its biological and clinical efficacy [37]. Additionally, the part of the plant used substantially determines the composition of the final product [38]. Generally, the hypocotyl section is used for human consumption and supplementation because it contains the highest levels of bioactive compounds, particularly glucosinolates and macamides [39]. Glucosinolates have been proposed as chemical markers of maca quality and authenticity [40]. In addition, the phytochemical profile of *L. meyenii* includes a wide range of secondary metabolites, including alkaloids, flavonoids, sterols, and polysaccharides, which may contribute to its biological activity [35]. Among these, macamides and macaenes have been proposed as characteristic compounds of *L. meyenii*, due to their potential neuroprotective and anti-fatigue effects [41]. Furthermore, glucosinolates and their isothiocyanate derivatives have been linked to antioxidant and anti-inflammatory properties [42]. In addition, polyphenols and flavonoids may enhance the antioxidant capacity of *L. meyenii* by scavenging reactive oxygen species (ROS) and modulating redox-sensitive signaling pathways [43]. These combined phytochemical interactions may partially explain the physiological effects attributed to *L. meyenii*, particularly in the context of exercise-induced oxidative stress and muscle damage.

**Figure 1:**
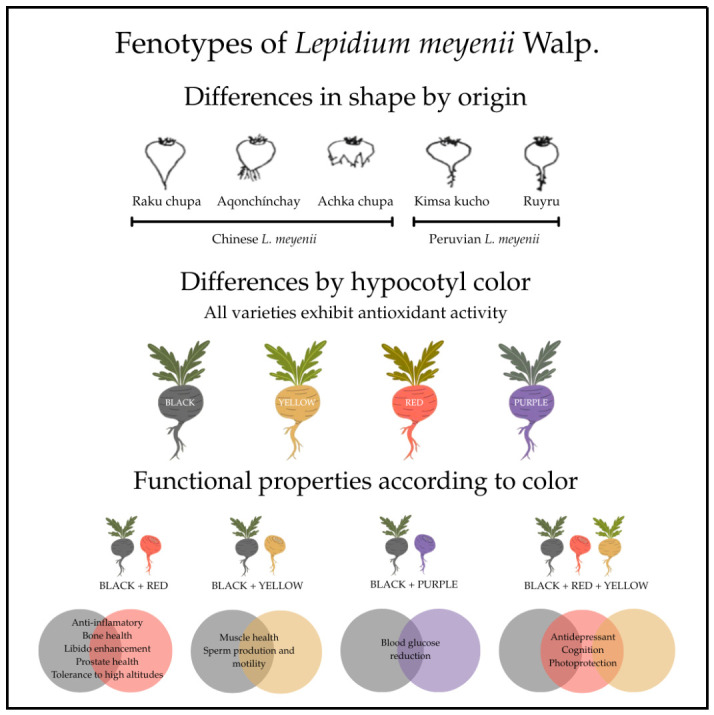
Phenotypes of *L. meyenii*.

From a phenotypic perspective, up to 17 different *L. meyenii* colors have been described. However, the most widely studied and used colors are purple, red, black, white, gray, and yellow, which differ in their profiles of bioactive compounds and potential functional effects, as shown in Figure 1 [37].

Also, the interest in investigating the effects of *L. meyenii* on other areas has been increasing. Recent studies have demonstrated that *L. meyenii* exhibits antihypertensive, anti-inflammatory, neuroprotective, antithrombotic, cardioprotective, and other beneficial properties [28]. Regardless of extraction type, botanical variety, or specific phytochemical profile, this study focused exclusively on *L. meyenii* products classified as *L. meyenii*.

At the same time, scientific evidence indicates that *L. meyenii* supplementation can confer benefits during physical activity, exercise, and sports, mainly by attenuating cellular oxidative stress [44,45]. At the molecular level, *L. meyenii* has been shown to increase Nrf2 expression [46], thereby promoting transcription of mRNAs encoding key antioxidant enzymes, such as superoxide dismutase (SOD), glutathione peroxidase (GPx), and catalase (CAT) [46]. The activation of these endogenous antioxidant systems contributes to the reduction in ROS and malondialdehyde (MDA) levels, thereby mitigating exercise-induced oxidative stress [47]. Specifically, MDA is a final product of ROS-induced lipid peroxidation and is therefore widely used as a marker of cellular oxidative stress [48]. Consistent with this, *L. meyenii* consumption has been associated with reductions in BUN levels and decreased LDH and CK activity [49,50,51], suggesting a protective effect against muscle damage. These effects could be related to the ability of *L. meyenii* to modulate glycogen and amino acid metabolism, optimize the production of adenosine triphosphate (ATP), and promote the elimination of lactate in skeletal muscle during physical exertion [49].

Although evidence suggests that *Lepidium meyenii* may mitigate the effects of EIMD, current findings are fragmented, heterogeneous, and inconsistent regarding its impact on blood biomarkers of muscle damage and catabolic stress (CK, LDH, and BUN). To date, no systematic review has quantified the magnitude of these effects or explored factors such as dose–response relationships. Therefore, the objective of this study was to conduct a qualitative review and estimate the effect size (ES) of *L. meyenii* supplementation on CK, LDH, and BUN in animals subjected to physical stress, considering publications up to April 2026. In addition, given that the interventions included differed in composition and dosage, we investigated whether the type of preparation (whole/raw extract versus purified/isolated compound) or the administered dose accounted for the observed heterogeneity among the studies.

## 2. Materials and Methods

This systematic review and meta-analysis were conducted in accordance with established methodological guidelines for systematic reviews and meta-analyses [52]. The review includes only preclinical studies in which EIMD was induced through experimentally induced exercise protocols. To assess the potential risk of bias in the included studies, the Collaborative Approach for Meta-Analysis and Review of Animal Data from Experimental Studies (CAMARADES) tool was applied [53]. The review protocol was registered in PROSPERO under registration number CRD42025630693.)

### 2.1. Selection Criteria

The selection of studies was based on an established systematic review methodology [52] and was structured using the PICOS approach, which defined (i) the population (healthy animals subjected to exercise protocols designed to induce EIMD), (ii) the intervention (*L. meyenii* supplementation on blood biomarkers post-exertion muscle damage and protein degradation), (iii) the comparators (a control group without supplementation and an experimental group with *L. meyenii* supplementation, with pre and post-intervention assessments), (iv) outcomes (positive or negative changes in blood biomarkers of muscle damage and protein degradation measured after an exercise intervention) and (v) the quasi-experimental study design with control and experimental groups. Studies that did not meet these criteria were excluded from the review and meta-analysis. Any disagreements among the authors during the search process were resolved through discussion until a consensus was reached.

### 2.2. Search Strategy and Information Sources

Relevant articles were identified through searches in Web of Science (WoS), PubMed, Scopus, MEDLINE, and SPORTDiscus, covering publications from the databases’ inception through April 2026. The search included studies published from inception to April 2026. Keywords such as [(“*Lepidium meyenii* walp” OR “Maca” OR “Macamides” OR “*Lepidium peruvianum*” OR “Ginseng Andean” OR “Ginseng Peruvian” OR “Ayak Chichira” OR “Ayak Willku” OR “Black maca” OR “Red maca” OR “Yellow maca” OR “Maca polysaccharide” OR “Maca powder” OR “Maca extract” OR “Glucosinolates of maca” OR “Peruvian maca”) AND (“muscle damage” OR “creatine kinase” OR “lactate dehydrogenase” OR “blood urea nitrogen”)] were used, combined with Boolean operators (AND/OR). The RefWorks bibliographic manager was used to manage the search results. The initial search was conducted by two authors (J.R.R., A.H.O.), and a subsequent evaluation of the studies for inclusion was performed by three authors (J.R.R., A.H.O., and G.B-F.).

### 2.3. Data Extraction

Data obtained from the selected studies included the author, year, publication type, sample size, type of intervention, independent and dependent variables (focusing on *L. meyenii* supplementation, muscle damage, and protein degradation), outcomes, and characteristics of the control and experimental groups. All the results obtained corresponded to biomarkers measured following protocols for exercise-induced muscle damage in animal models. The research team conducted the data collection process independently. In cases where reports were incomplete, the study’s original authors were contacted by mail to obtain the necessary metrics. When relevant data were missing, the corresponding authors were contacted by email to request the necessary information. In cases where no response was received, data were manually extracted from the figures provided in the publications using pixel-based estimation. Discrepancies between authors were resolved through discussion. The doses and their ranges used in the present study were previously established by Chen et al. [54]. The studies included in the systematic review and meta-analysis were classified into the following dose ranges: low (<400 mg/kg), medium (400–800 mg/kg), and high (>800 mg/kg) [44,45,54]. These categories were adopted to facilitate comparisons between studies and do not represent standardized or recommended dosage ranges for human supplementation.

### 2.4. Risk of Publication Bias Among Studies

Publication bias was assessed exclusively in the studies included in the meta-analysis using Egger’s regression test, with a statistical significance level of *p* ≤ 0.05 [55]. In addition, funnel plots were generated to visually assess the combined effects, and Egger’s test was used to assess potential publication bias.

### 2.5. Assessment of Methodological Quality and Risk of Bias in Individual Studies

In animal studies, methodological rigor and potential sources of bias were assessed using the CAMARADES checklist [53]: This tool considers ten domains: publication following peer review, control of ambient temperature, random assignment, concealment of group assignment, blinded assessment of results, avoidance of anesthetics with intrinsic protective effects, use of appropriate animal models, sample size estimation, compliance with animal welfare standards, and disclosure of conflicts of interest. The studies were classified into three categories: low quality (1–4 points), moderate quality (5–7 points), and high quality (8–10 points).

### 2.6. Statistical Analysis and Synthesis of Results

The systematic review findings were organized in an Excel spreadsheet to record primary variables, including study objectives, participant characteristics, intervention types, results, and measures of effect. The data were transferred to R statistical software (version 4.5.0) for the meta-analysis, using specialized packages such as meta, metafor, and robumeta (version 4.5.0).

The analysis of the effects of *L. meyenii* supplementation on blood biomarkers of muscle damage (including CK and LDH) and blood biomarkers of indirect protein metabolism and systemic stress (BUN) was standardized using standardized mean differences (SMDs), accompanied by 95% confidence intervals.

When studies included multiple experimental groups with a single control group, the effect sizes were not treated as independent but as statistically dependent. For this reason, robust variance estimation (RVE) was applied, following Tipton’s methodology [56], or multilevel multivariate analysis (MLMA) models were used, with the rma.mv function in R. The choice between these approaches depended on the number of available effect sizes and the hierarchical structure of the data.

Heterogeneity between studies was assessed using the *I*^2^ and τ^2^ statistics [57]. For interpreting *I*^2^, conventional criteria were used: low (25–50%), moderate (50–75%), and high (>75%). Whenever feasible, subgroup analyses were performed by dose level (low, medium, high) to examine potential effects related to the amount of maca administered.

The primary outcomes by biomarker and dose category were estimated using multilevel models (rma.mv) with robust variance estimation (RVE), employing a CR2 correction and Satterthwaite’s degrees of freedom, with an assumed intra-cluster correlation between effects that share the same control group. In addition, an exploratory analysis was conducted by type of preparation, grouping the interventions into two mechanistically distinct categories: whole/raw extracts (maca powder, aqueous and fat-soluble extracts, and root preparations) and purified/isolated compounds (isolated polysaccharide and macamide fractions). In addition, a meta-regression was conducted with the type of formulation and the dose (on a natural logarithmic scale, mg/kg) as simultaneous moderators, in order to assess whether any of these factors accounted for heterogeneity. This analysis was applied only to LDH and BUN; it was not possible to do so for CK due to the lack of variation in dosage and the small number of studies. The type of preparation and the dose showed partial collinearity in this dataset; therefore, the independent contribution of each factor should be interpreted with caution.

## 3. Results

### 3.1. Studies Selection

**Figure 2:**
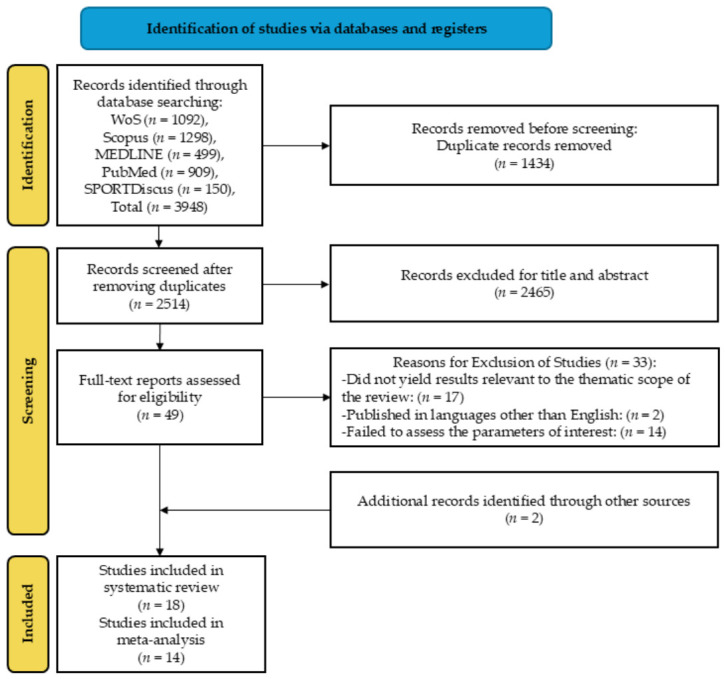
Search strategy and study selection.

The database search yielded 3948 studies, of which 1434 were identified as duplicates and therefore excluded. After reviewing the titles and abstracts, the full text of 49 studies was evaluated to determine their eligibility. Of these, 36 articles were excluded for failing to meet the inclusion criteria. In addition, 2 relevant studies were identified through other sources. Finally, 15 studies were included in the systematic review and 14 in the meta-analysis [29,31,49,50,51,54,58,59,60,61,62,63,64,65,66]. Figure 2 provides a detailed description of the search and selection process.

Table 1 details the characteristics of the studies, the doses of *L. meyenii* administered, and the results related to muscle damage and post-exertion protein degradation.

### 3.2. Assessment of Methodological Quality of Individual Studies

The results of the animal studies evaluated using the CAMARADES scale showed that nine studies [29,31,49,50,51,54,61,62,65] obtained a total score of 7 stars out of a possible 10, indicating moderate methodological quality. These studies met the criteria of peer-reviewed publication, temperature control, random assignment to treatment or control, use of anesthetics without intrinsic properties, use of appropriate animal models, and compliance with animal welfare regulations. However, no mention was made of allocation concealment procedures, blinded outcome assessment, or sample size calculations.

Likewise, He et al. [58] and Zheng et al. [59] obtained an overall score of 5 stars, meeting the criteria of peer-reviewed publication, random assignment to treatment or control, use of anesthetics with no intrinsic properties, use of appropriate animal models, and compliance with animal welfare regulations. However, they failed to meet the requirements for temperature control, allocation concealment, blinded evaluation of results, sample size calculation, and absence of conflicts of interest. Zheng et al. [60] achieved a score of 6 stars, demonstrating slightly better performance than He et al. [58] and Zheng et al. [59] as they met an additional criterion. Despite this, the three studies are of moderate quality. The methodological quality analysis of all the studies can be downloaded from the following link: https://doi.org/10.6084/m9.figshare.32595393 (accessed on 8 June 2026).

### 3.3. Meta-Analysis

Among the 15 selected studies, 14 included randomized controlled trials, pre-test and post-test designs, experimental and control groups, and meta-analyzable outcomes [29,31,49,50,51,54,58,59,60,61,62,63,64,65]. These 14 studies, all performed on animal groups, were meta-analyzed using two muscle damage markers: CK [50,62,66], LDH [29,31,50,51,54,58,59,60,61,65,66,67], and a biomarker associated with protein degradation: BUN [29,31,49,50,54,60,61,62,63,64,65,66]. In all included studies, the control group served as a reference for each experimental group within the same study. When there were multiple experimental groups, each comparison was constructed relative to a shared control group, yielding dependent effect sizes at the intrastudy level. To properly account for these within-study dependencies, the main results presented in Table 2 were obtained using robust variance estimation (RVE) and multilevel models (MLMA). These approaches allow for multiple comparisons within a study by adjusting for the shared-control design and avoiding the underestimation of standard errors, which would otherwise overstate statistical significance.

For visualization purposes, funnel plots and forest diagrams were generated using classical random effects models. They should be interpreted with caution, as they do not explicitly account for intra-study dependencies arising from shared control groups. Consequently, the number of effect sizes shown in the supplementary forest plots may differ slightly from those reported in the main results of the meta-analysis.

### 3.4. Evaluation of the Quality of Evidence (GRADE)

The quantitative analysis was completed by evaluating the certainty of the evidence using the GRADE (Grading of Recommendations Assessment, Development, and Evaluation) system. This evaluation considered aspects such as methodological quality, consistency of the results, precision of the estimated effects, potential for bias, and clinical applicability of the included studies. The results showed ‘Very low’ certainty for CK, LDH, and BUN, primarily due to high heterogeneity, imprecise estimates—with confidence intervals that include zero—and a potential risk of bias among the included studies. The certainty of evidence for each biomarker analyzed is detailed in Table 3.

### 3.5. Effect of L. meyenii on Creatine Kinase

Three studies were considered for this analysis [50,58,60]. Since some studies included multiple experimental groups with a single control group, all extracted comparisons were meta-analyzed as dependent variables. To analyze this dependency, RVE and MLMA estimates were calculated. Specifically, the study of He et al. [58] provided three comparisons (a, b, and c), Tang et al. [50] provided three comparisons (a, b, and c), while Zheng et al. [60] provided five comparisons (a, b, c, d, and e). In total, 11 effect sizes from three studies were included to estimate the effect of *L. meyenii* on CK, all of which involved low doses.

**Figure 3:**
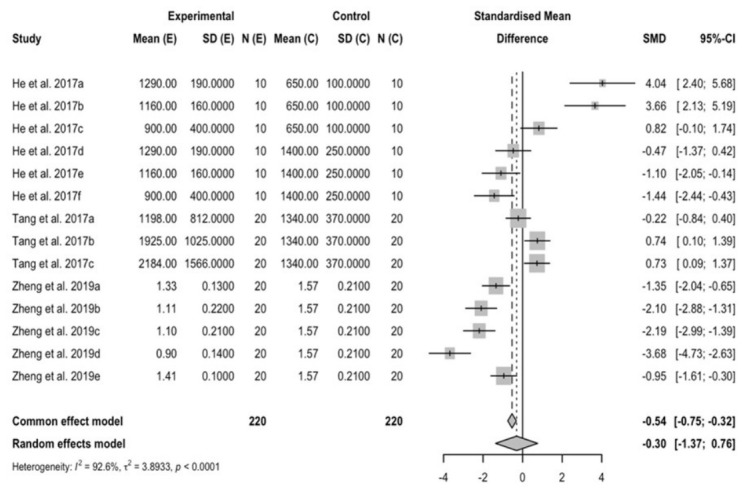
Forest plot comparing the effects of *L. meyenii* on CK blood concentrations [50,58,60].

Figure 3 shows that *L. meyenii* had no significant effect on the CK (SMD = 0.29; 95% CI −5.45 to 6.03; *p* = 0.847), with very high heterogeneity (*I*^2^ = 97.4%). Since this analysis included only three studies, the robust variance estimate is unreliable, and the result should be interpreted with caution.

### 3.6. Effect of L. meyenii on Lactate Dehydrogenase

Ten studies were considered for this analysis [31,49,50,51,54,58,59,60,61,65,67]. Specifically, Chen et al. [54] provided nine comparisons; Choi et al. [51] two; He et al. [58] three; Li et al. [61] four; Liu et al. [65] two; Tang et al. [50] three; Yang et al. [31] six; Zheng et al. [59] one; Zheng et al. [60] five; Zhu et al. [49] two; and Zhu et al. [29] four. In total, 41 effect sizes from 10 studies were included for LDH.

**Figure 4:**
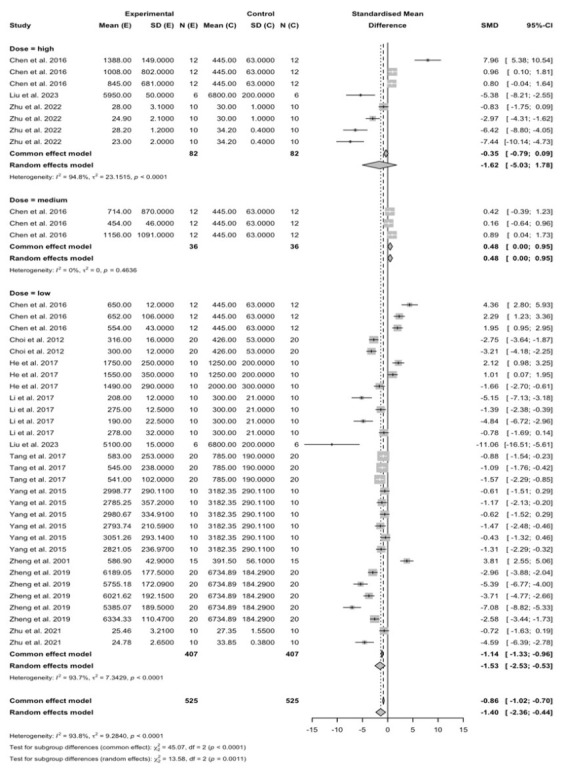
Forest plot comparing the effects of *L. meyenii* on LDH blood concentrations [29,31,49,50,54,58,59,60,61,65].

Figure 4 shows that the overall analysis, corrected for dependency, did not reveal a significant effect of *L. meyenii* on LDH (SMD = −1.37; 95% CI −3.34 to 0.59; *p* = 0.148). In the subgroup analyses, no dose category reached statistical significance (low: SMD = −1.21; 95% CI −3.82 to 1.40; *p* = 0.317; high: SMD = −1.24; 95% CI −11.62 to 9.15; *p* = 0.652); the moderate-dose subgroup consisted of a single study and could not be assessed. Heterogeneity was very high in all cases (*I*^2^ > 97%).

### 3.7. Effect of L. meyenii on Blood Urea Nitrogen

Eleven studies were considered for this analysis [29,31,49,50,59,60,61,62,63,64,68]. Since several of these studies included more than one experimental group, multiple comparisons were extracted from the same study and analyzed using MLMA. Specifically, Chen et al. [54] provided nine comparisons; Li et al. [61] four; Li et al. [63] three; Li et al. [64] three; Li et al. [62] three; Liu et al. [65] two; Tang et al. [50] three; Yang et al. [31] six; Zheng et al. [60] five; Zhu et al. [49] two; and Zhu et al. [29] eight.

**Figure 5:**
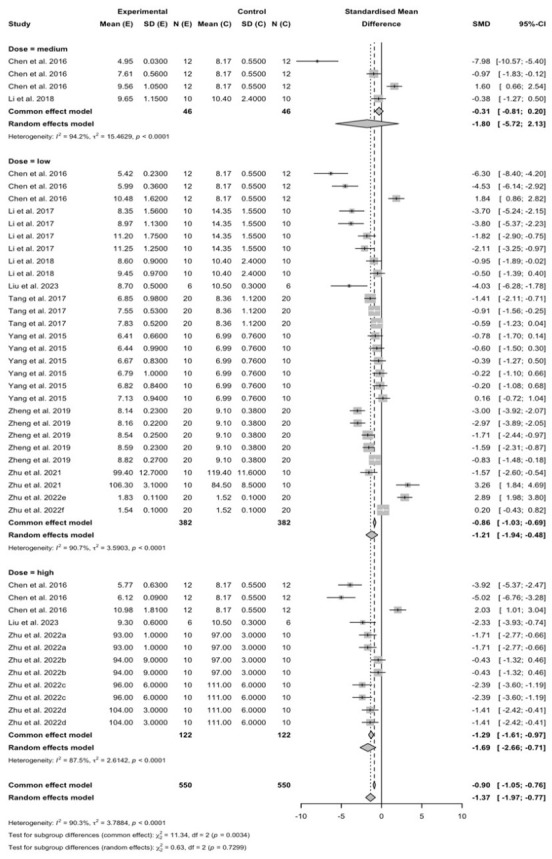
Forest plot comparing the effects of *L. meyenii* on BUN blood concentrations [31,49,50,54,60,61,62,63,64,65,67].

In total, 43 effect sizes from 11 studies were included for BUN; of these, 26 corresponded to low doses, 6 to moderate doses, and 11 to high doses. Figure 5 shows that, after adjusting for confounding factors, *L. meyenii* had no significant effect on BUN (SMD = −0.37; 95% CI −2.16 to 1.42; *p* = 0.657), and no dose subgroup was significant (low: SMD = −0.68; 95% CI −2.67 to 1.31; *p* = 0.455; moderate: SMD = −0.68; 95% CI −5.44 to 4.09; *p* = 0.641; high: SMD = 1.24; 95% CI −6.04 to 8.52; *p* = 0.660). Heterogeneity was very high (*I*^2^ > 97%).

### 3.8. Exploratory Analysis by Type of Preparation and Dose

To determine whether the effect of *L. meyenii* depended on the type of preparation or the dose rather than on a generic effect, two complementary exploratory analyses were conducted (Table 4). In the subgroup analysis by preparation, purified/isolated compounds were associated with lower LDH (SMD = −2.04; 95% CI −3.97 to −0.12; seven studies), whereas whole/crude extracts showed no effect (SMD = −0.09; 95% CI −2.70 to 2.52; four studies); for BUN, no category was significant, and both preparations pointed in opposite directions. However, when both preparation and dose were simultaneously included in a meta-regression, neither moderator proved significant—neither for LDH (preparation: *β* = −1.95; 95% CI −5.83 to 1.93; *p* = 0.151; dose: *β* = −0.08; 95% CI −0.67 to 0.51; *p* = 0.737; omnibus test *p* = 0.319) nor for BUN (preparation: *β* = 5.38; *p* = 0.352; dose: *β* = 0.42; 95% CI −0.10 to 0.94; *p* = 0.094; omnibus test *p* = 0.381), and heterogeneity remained largely unexplained. The apparent association of the subgroup of purified compounds, therefore, did not hold up when adjusted for dose, with which it remains collinear. In CK, this analysis was not possible due to the lack of dose variation and the small number of studies. Taken together, these exploratory results indicate that neither the type of preparation nor the dose robustly explains the observed heterogeneity.

## 4. Discussion

This study examined the impact of *L. meyenii* supplementation on indicators of muscle damage and protein breakdown following physical exercise. After appropriately modeling the relationship between effect sizes, supplementation with *L. meyenii* was not associated with significant changes in LDH, BUN, or CK in animals subjected to physical stress. Exploratory analyses by preparation type and dose did not identify any moderators that could explain the high heterogeneity observed. However, the high degree of heterogeneity observed among the results suggests that these findings should be interpreted with caution. Differences in exercise protocols, the timing of biomarker assessment, the duration of supplementation, the maca phenotype, extraction methods, and the composition of bioactive compounds may have contributed to the variability across studies. This variability could partly explain the inconsistency in the effects observed across different biomarkers, particularly the absence of significant changes in CK. Consequently, the results of this study should be viewed as exploratory and hypothesis-generating rather than as confirmatory evidence.

### 4.1. Effect of L. meyenii on Creatine Kinase

During muscle contraction, various processes occur, including activation of oxidative enzymes, increased release and oxidation of catecholamines, electron leakage in the electron transport chain, and an increased inflammatory response [69]. If the effort increases in intensity, anaerobic processes for synthesizing ATP take precedence [70]. In these processes, specifically the ATP-PC cycle, CK is crucial as it provides immediate energy to the cell during the first seconds of muscle contraction in high-intensity exercise [71,72]. However, when the external mechanical load exceeds the contractile capacity of skeletal muscle, EIMD is generated [73]. This damage results in rupture of the cell plasma membrane and, consequently, the release of cellular components into the bloodstream, including CK [74] and specific ROS [75], such as protein carbonyls, MDA, and 8-hydroxy-2′-deoxyguanosine (8-OHdG) [76]. The results of the present study showed no significant effect after *L. meyenii* supplementation [50,58,60]. Preclinical evidence indicates that *L. meyenii* consumption may be associated with decreased ROS [45]. Future studies should consider measuring CK at multiple time points following exercise, for example, at 12, 24, and 72 h [77], to gain a more comprehensive understanding of its behavior. It would also be important to examine the effect of different doses of *L. meyenii* on this biomarker, which would allow for the analysis of a potential dose–response relationship and contribute to a better understanding of its physiological effects under conditions of exercise-induced stress.

### 4.2. Effect of L. meyenii on Lactate Dehydrogenase

LDH is an enzyme belonging to the group of oxidoreductases, which facilitates the interconversion between pyruvate and lactate, allowing the reversible conversion between nicotinamide adenine dinucleotide (NAD) and its reduced form, NADH, to maintain intracellular energy homeostasis [15]. The evidence is conclusive that LDH levels increase in response to physical stress or muscle injury [78]. It has also been demonstrated that LDH activity and concentrations are highly dependent on the individual’s level of training; for example, for the same level of effort, higher LDH concentrations have been observed in less-trained individuals [79]. After adjusting for the effect size dependency, this meta-analysis did not find a significant reduction in LDH following supplementation with *L. meyenii* (SMD = −1.37; *p* = 0.148), and no dose subgroup was significant. Although a subgroup analysis suggested a possible reduction associated with purified compounds, this association was not maintained when adjusting simultaneously for preparation and dose [54]. A study analyzed the effect of supplementation with crude extract and purified extract of *L. meyenii*-derived macamides on exercise-induced muscle damage in rats subjected to a swim-to-exhaustion test, observing that macamide administration was associated with decreased LDH and CK levels, as well as attenuation of histopathological signs of muscle damage, including necrosis, pyknosis, and karyorrhexis in skeletal muscle tissue [60]. Therefore, the current evidence is insufficient to establish a clear link between the antioxidant properties of *L. meyenii* and changes in LDH [31,59].

### 4.3. Effects of L. meyenii on Blood Urea Nitrogen

BUN is a metabolic product of protein degradation that occurs when ATP levels in the musculoskeletal cell are insufficient to sustain muscle contraction [80]. This results in a leakage of calcium ions from the extracellular space into the intracellular space, which in turn triggers an increase in the activity of intracellular proteolytic enzymes [81]. Additionally, it has been observed that exercise induces physical stress, which in turn increases cortisol levels [82]. This hormone promotes the breakdown of proteins into amino acids [83] and induces oxidative stress [84]. These amino acids are then used as an energy source for other processes, such as gluconeogenesis [85]. The amino group removed during this decomposition is converted to urea, which is reflected in the increase in the BUN [86]. For this reason, BUN is widely used as an index of protein catabolism, kidney function and hydration status [87]. The observed changes in BUN should be interpreted as a response to exercise-induced metabolic stress rather than as evidence of reduced muscle damage.

This study did not find a significant reduction in BUN following supplementation with *L. meyenii* after accounting for the effect size dependence (SMD = −0.37; *p* = 0.657). In fact, some preparations, such as high-dose maca root, were associated with an increase in BUN, reinforcing the notion that changes in this biomarker should be interpreted as a response to exercise-induced metabolic stress and not as evidence of reduced muscle damage. The high heterogeneity among studies [29,31,49,50,54,60,61,62,63,64,65] supports this interpretation. Despite the encouraging results, the physiological pathways by which *L. meyenii* could reduce BUN levels are not fully described. One possible hypothesis is that its antioxidant effects help mitigate inflammation and oxidative stress [45], which favors EIMD-induced muscle recovery [88]. However, due to the limited number of investigations that studied the effect of *L. meyenii* on blood BUN concentrations, more studies covering these variables are needed.

### 4.4. Limitations

Although the results presented in the systematic review and meta-analysis suggest a potential effect and provide a preliminary understanding of the impact of *L. meyenii* on blood biomarkers of post-exertional muscle damage and protein degradation, the following limitations need to be considered: (a) Publication bias could not be reliably assessed due to the small number of studies per outcome, so it cannot be ruled out; (b) The included interventions differ considerably in their phytochemical profile, concentration of bioactive compounds, and bioavailability. To address this heterogeneity, exploratory analyses were conducted by preparation type, and a meta-regression was performed that included preparation and dose; however, none of these factors explained the observed variability, and the partial collinearity between preparation and dose prevented the complete separation of their contributions. Therefore, the results do not reflect the effect of a specific preparation but rather a general estimate of heterogeneous interventions; (c) To interpret the ES of *L. meyenii* on post-exertion CK levels, it should be considered that CK evaluations immediately post-exercise were included, as well as only the effect of low doses of *L. meyenii* were evaluated, and (d) The GRADE evaluation indicated that the certainty of the evidence was very low for all assessed outcomes, mainly due to high heterogeneity, risk of bias, and the limited number of studies in some doses. These limitations restrict the generalizability of the results and underscore the need for future research with greater methodological rigor and larger sample sizes. (e) It was not possible to stratify by *L. meyenii* phenotype (color) because most studies did not report this information, nor was it possible to explore cross-subgroup analyses (e.g., dose-by-biomarker interactions) due to an insufficient number of effects per cell, which would have resulted in unstable estimates; In CK, in particular, dose analysis and meta-regression were not possible due to the lack of dose variation and the small number of studies. Furthermore, all studies included in this review were conducted in animal models exposed to experimentally induced physical stress protocols. Therefore, the applicability of these findings to humans, athletes, or clinical populations remains uncertain and should be interpreted with caution.

## 5. Conclusions

In conclusion, once the relationship between effect sizes was properly modeled, the available evidence does not support a robust pooled effect of *L. meyenii* supplementation on blood biomarkers of muscle damage (CK, LDH) or protein catabolism (BUN) in animal models subjected to physical stress. The high heterogeneity was not robustly explained by the type of preparation or the dose, and some preparations were even associated with an increase in BUN. Taken together, these results do not support a direct protective effect of *L. meyenii* supplementation against exercise-induced muscle damage.

For this reason, these findings should be interpreted with caution, especially given the preclinical nature of the included studies and the heterogeneity observed among them. Randomized controlled clinical trials are needed to more accurately determine the effect of *L. meyenii* on blood biomarkers associated with muscle damage and post-exercise protein breakdown, as well as systematizing the use of standardized and well-characterized preparations, which allow for a more consistent evaluation of the effects of their bioactive compounds.

## 6. Prospects for *L. meyenii* Supplementation in Humans

This review focused its search on studies demonstrating the effect of *L. meyenii* on blood biomarkers of muscle damage and post-exertion protein degradation. Both the search and the results focused on evidence from animal models. However, during the search, three human studies were identified that evaluated the same outcomes [89,90,91]. Overall, the results of these studies were mixed and inconclusive. For example, Lee et al. [89] observed a significant decrease in CK levels in swimmers using fins, whereas no significant differences were found in other sports such as shooting and racquet sports. Similarly, Liu et al. [90] observed a significant reduction in LDH, but no change in CK, in healthy men undergoing a strenuous endurance test. On the other hand, Honma et al. [91] reported non-significant differences in adult women. Overall, human data are limited and inconsistent and do not allow conclusions to be drawn regarding the effect of *L. meyenii* on biomarkers of exercise-induced muscle damage. This search did not find any information regarding the effects of *L. meyenii* supplementation on BUN. The promising results of *L. meyenii* supplementation in animals and the lack of data in humans suggest the need for randomized controlled clinical trials to clarify its potential role in mitigating muscle damage and post-exertion protein degradation.

## Figures and Tables

**Table 1:** Characteristics of the studies that connect *L. meyenii* with post-exertion muscle damage blood biomarkers.

Blood Markers of Post-Exertion Muscle Damage							
Authors	Objective	Participants or Sample	Independent Variable	Dependent Variable	Supplementation Protocol	Results	Effect
Bilal et al. [66]	To investigate the effects of maca on serum indices and metabolic responses in racehorses	Racehorses: EG1 (*n* = 6) EG2 (*n* = 6) CG (*n* = 6)	EG: MPB CG: Basal diet	CK and LDH	Maca root extract powder: EG1: Basal diet + 50 g·day EG2: Basal diet + 75 g·day CG: Basal diet	CK (IU·L): EG1 post-test = 117.83 vs. CG post-test = 160.17 EG2 post-test = 176.80 vs. CG post-test = 160.17 LDH (IU·L): EG1 post-test = 288.80 vs. CG post-test = 272.83 EG2 post-test = 274.81 vs. CG post-test = 272.83	CK (IU·L): EG1 post test vs. CG ↓ EG2 post test vs. CG ↑ LDH (IU·L): EG1 post-test vs. CG ↑ EG2 post-test vs. CG ↑
Chen et al. [54]	To investigate the effects of *L. meyenii* (maca) on hypoxia tolerance and fatigue relief and to determine its active constituents	Mice: EG1 (*n* = 12) EG2 (*n* = 12) EG3 (*n* = 12) EG4 (*n* = 12) EG5 (*n* = 12) EG6 (*n* = 12) EG7 (*n* = 12) EG8 (*n* = 12) EG9 (*n* = 12) CG (*n* = 12)	EG: MPB, ME and MWP CG: PL	LDH	MP: EG1 (high-dose): 1 g·kg^−1^ EG2 (medium-dose): 0.5 g·kg^−1^ EG3 (low-dose): 0.1 g·kg^−1^ MAE: EG4 (high-dose): 1 g·kg^−1^ EG5 (medium-dose): 0.5 g·kg^−1^ EG6 (low-dose): 0.1 g·kg^−1^ MWP: EG7 (high-dose): 1 g·kg^−1^ EG8 (medium-dose): 0.5 g·kg^−1^ EG9 (low-dose): 0.1 g·kg^−1^ CG: distilled water	LDH (IU·L): MP groups: EG1 = 1388 ± 149 vs. CG = 445 ± 63; *p* < 0.01 EG2 = 714 ± 870 vs. CG = 445 ± 63; *p* > 0.05 EG3 = 650 ± 12 vs. CG = 445 ± 63; *p* > 0.05 MAE groups: EG4 = 1008 ± 802 vs. CG = 445 ± 63; *p* > 0.05 EG5 = 454 ± 46 vs. CG = 445 ± 63; *p* > 0.05 EG6 = 652 ± 106 vs. CG = 445 ± 63; *p* > 0.05 MWP groups: EG7 = 845 ± 681 vs. CG = 445 ± 63; *p* > 0.05 EG8 = 1156 ± 1091 vs. CG = 445 ± 63; *p* > 0.05 EG9 = 554 ± 43 vs. CG = 445 ± 63; *p* > 0.05	LDH (IU·L): MP groups: EG1 vs. CG ↑ EG2 vs. CG ↔ EG3 vs. CG ↔ MAE groups: EG4 vs. CG ↔ EG5 vs. CG ↔ EG6 vs. CG ↔ MWP groups: EG7 vs. CG ↑ EG8 vs. CG ↔ EG9 vs. CG ↔
Choi et al. [51]	To investigate the effect of standardized LME obtained by supercritical fluid extraction of maca on swimming endurance capacity, serum biochemical parameters, and antioxidant status in a weight-loaded forced swimming rat model	Mice: EG1 (*n* = 20) EG2 (*n* = 20) CG (*n* = 20)	EG1 and EG2: LME CG: PL	LDH	LME: EG1: 30 mg·10 mL·kg^−1^ EG2: 100 mg·10 mL·kg^−1^ CG:10 mL·kg^−1^ sterile water	LDH (U·L): EG1 = 316 ± 16 vs. CG = 426 ± 53; *p* > 0.05 EG2 = 300 ± 12 vs. CG = 426 ± 53; *p* < 0.05	LDH (U·L): EG1 vs. CG ↔ EG2 vs. CG ↓
He et al. [58]	To investigate the effects of MP on oxidative damage induced by exhaustive swimming exercise using rat models	Mice: EG1 (*n* = 10) EG2 (*n* = 10) EG3 (*n* = 10) CG1 (*n* = 10) CG2 (*n* = 10)	EG: MP CG: PL	CK and LDH	MP EG1: exercise + 50 mg·kg^−1^ EG2: exercise + 100 mg·kg^−1^ EG3: exercise + 200 mg·kg^−1^ CG1: sedentary + distilled water CG2: exercise + distilled water	CK (U·L): EG1 = 1290 ± 190 vs. CG1= 650 ± 100; *p* < 0.05 EG1 = 1290 ± 190 vs. CG2= 1400 ± 250; *p* < 0.05 EG2 = 1160 ± 160 vs. CG1= 650 ± 100; *p* < 0.05 EG2 = 1160 ± 160 vs. CG2= 1400 ± 250; *p* < 0.05 EG3 = 900 ± 400 vs. CG1= 650 ± 100; *p* < 0.05 EG3 = 900 ± 400 vs. CG2= 1400 ± 250; *p* < 0.05 CG1 = 650 ± 100 vs. CG2= 1400 ± 250; *p* < 0.05 LDH (IU·L): EG1 = 1750 ± 250 vs. CG1 = 1250 ± 200; *p* < 0.05 EG1 = 1750 ± 250 vs. CG2 = 2000 ± 300; *p* < 0.05 EG2 = 1550 ± 350 vs. CG1 = 1250 ± 200; *p* < 0.05 EG2 = 1550 ± 350 vs. CG2 = 2000 ± 300; *p* < 0.05 EG3 = 1490 ± 290 vs. CG1 = 1250 ± 200; *p* < 0.05 EG3 = 1490 ± 290 vs. CG2 = 2000 ± 300; *p* < 0.05 CG1 = 1250 ± 200 vs. CG2= 2000 ± 300; *p* < 0.05 CG1 = 188 ± 34 vs. CG2= 114 ± 21; *p* < 0.05	CK (U·L): EG1 vs. CG1 ↑ EG1 vs. CG2 ↓ EG2 vs. CG1 ↑ EG2 vs. CG2 ↓ EG3 vs. CG1 ↑ EG3 vs. CG2 ↓ CG1 vs. CG2 ↓ LDH (IU·L): EG1 vs. CG1 ↑ EG1 vs. CG2 ↓ EG2 vs. CG1 ↑ EG2 vs. CG2 ↓ EG3 vs. CG1 ↑ EG3 vs. CG2 ↓ CG1 vs. CG2 ↓ CG1 vs. CG2 ↓
Li et al. [61]	To isolate and characterize the purified MP and specify the anti-fatigue composition of MP	Mice: EG1 (*n* = 10) EG2 (*n* = 10) EG3 (*n* = 10) EG4 (*n* = 10) CG (*n* = 10)	EG: MP CG: PL	LDH	MP-1 EG1: 100 mg·kg^−1^ EG2: 20 mg·kg^−1^ MP-2 EG3: 100 mg·kg^−1^ EG4: 20 mg·kg^−1^ CG: saline solution	LDH (IU·L): EG1 = 208 ± 12 vs. CG = 300 ± 21; *p* < 0.05 EG2 = 275 ± 12.5 vs. CG = 300 ± 21; *p* < 0.05 EG1 = 190 ± 22.5 vs. CG = 300 ± 21; *p* < 0.05 EG1 = 278 ± 32 vs. CG = 300 ± 21; *p* <0.05	LDH (IU·L): EG1 vs. CG ↓ EG2 vs. CG ↓ EG3 vs. CG ↓ EG4 vs. CG ↓
Liu et al. [65]	To investigate the anti-fatigue capacity of NBH	Mice EG1 (*n* = 6) EG2 (*n* = 6) CG (*n* = 6)	EG: ME and NBH CG: PL	LDH	ME EG1: 1000 mg·kg^−1^ extract of maca NBH EG2: 10 mg·kg^−1^ CG: distilled water	LDH (IU·L): EG1 = 5950 ± 50 vs. CG = 6800 ± 200; *p* < 0.01 EG2 = 5100 ± 150 vs. CG = 6800 ± 200; *p* < 0.01	LDH (U·L): EG1 vs. CG ↓ EG2 vs. CG ↓
Tang et al. [50]	To investigate the antifatigue effect of MP was also evaluated by using a mouse weight-loaded swimming model to provide a theoretical basis and practical guidance for the comprehensive exploration of MP	Mice: EG1 (*n* = 20) EG2 (*n* = 20) EG3 (*n* = 20) CG (*n* = 20)	EG: MP CG: PL	LDH and CK	MP EG1: 100 mg·kg^−1^ EG2: 50 mg·kg^−1^ EG3: 25 mg·kg^−1^ CG: distilled water	LDH (U·L): EG1 = 583 ± 253 vs. CG = 785 ± 190; *p* > 0.05 EG2 = 545 ± 238 vs. CG = 785 ± 190; *p* < 0.05 EG3 = 541 ± 102 vs. CG = 785 ± 190; *p* < 0.01 CK (U·L): EG1 = 1198 ± 812 vs. CG = 1340 ± 370; *p* > 0.05 EG2 = 1925 ± 1025 vs. CG = 1340 ± 370; *p* > 0.05 EG3 = 2184 ± 1566 vs. CG = 1340 ± 370; *p* < 0.05	LDH (U·L): EG1 vs. CG ↔ EG2 vs. CG ↓ EG3 vs. CG ↓ CK (U·L): EG1 vs. CG ↔ EG2 vs. CG ↔ EG3 vs. CG ↑
Yang et al. [31]	To investigate the effects of macamides on endurance capacity and anti-fatigue properties in prolonged swimming mice	Mice: EG1 (*n* = 10) EG2 (*n* = 10) EG3 (*n* = 10) EG4 (*n* = 10) EG5 (*n* = 10) EG6 (*n* = 10) CG (*n* = 10)	EG: *N*-benzyllinoleamide, *N*-benzyloleamide and *N*-benzylpalmitamideCG: PL	LDH	*N*-benzyllinoleamide EG1: 12 mg·10 mL·kg^−1^ EG2: 40 mg·10 mL·kg^−1^ *N*-benzyloleamide EG3: 12 mg·10 mL·kg^−1^ EG4: 40 mg·10 mL·kg^−1^ *N*-benzylpalmitamide EG5: 12 mg·10 mL·kg^−1^ EG6: 40 mg·10 mL·kg^−1^ CG: distilled water	LDH (U·L): EG1 = 2998.77 ± 290.11 vs. CG = 3182.35 ± 290.11; *p* > 0.05 EG2 = 2785.25 ± 357.2 vs. CG = 3182.35 ± 290.11; *p* < 0.05 EG3 = 2980. 67 ± 334.91 vs. CG = 3182.35 ± 290.11; *p* > 0.05 EG4 = 2793.74 ± 210.59 vs. CG = 3182.35 ± 290.11; *p* < 0.05 EG5 = 3051.26 ± 293.14 vs. CG = 3182.35 ± 290.11; *p* > 0.05 EG6 = 2821.05 ± 236.97 vs. CG = 3182.35 ± 290.11; *p* < 0.05	LDH (U·L): EG1 vs. CG ↔ EG2 vs. CG ↓ EG3 vs. CG ↔ EG4 vs. CG ↓ EG5 vs. CG ↔ EG6 vs. CG ↓
Zheng et al. [59]	To investigate the activity of energy enhancement of aqueous extracts from roots of Maca on the behavior in mice using FST	Mice: EG (*n* = 15) CG (*n* = 15)	EG: MacaForceAQ-2 CG: PL	LDH	MacaForce AQ-2 EG: 40 mg·kg^−1^ CG: 10% ethanol/water solution	LDH (U·100 mL) EG = 586.9 ± 42.9 vs. CG = 391.5 ± 56.1; *p* < 0.01	LDH (U·100 mL) EG vs. CG ↑
Zheng et al. [60]	To investigate the effect of two macamides extracts on attenuating muscle damage	Mice: EG1 (*n* = 20) EG2 (*n* = 20) EG3 (*n* = 20) EG4 (*n* = 20) EG5 (*n* = 20) CG (*n* = 20)	EG: CME, PME, and Maca tablet CG: PL	LDH and CK	CME EG1: 30 mg·kg^−1^ EG2: 120 mg·kg^−1^ PME EG3: 8 mg·kg^−1^ EG4: 32 mg·kg^−1^ Maca tablet EG5: 165 mg·kg^−1^ CG: aqueous solution	LDH (U·L) EG1 = 6189.05 ± 177.50 vs. CG = 6734.89 ± 184.29; *p* < 0.05 EG2 = 5755.18 ± 172.09 vs. CG = 6734.89 ± 184.29; *p* < 0.05 EG3 = 6021.62 ± 192.15 vs. CG = 6734.89 ± 184.29; *p* < 0.05 EG4 = 5385.07 ± 189.50 vs. CG = 6734.89 ± 184.29; *p* < 0.05 EG5 = 6334.33 ± 110.47 vs. CG = 6734.89 ± 184.29; *p* > 0.05 CK (U·mL): EG1 = 1.33 ± 0.13 vs. CG = 1.57 ± 0.21; *p* < 0.05 EG2 = 1.11 ± 0.22 vs. CG = 1.57 ± 0.21; *p* < 0.05 EG3 = 1.10 ± 0.21 vs. CG = 1.57 ± 0.21; *p* < 0.05 EG4 = 0.90 ± 0.14 vs. CG = 1.57 ± 0.21; *p* < 0.05 EG5 = 1.41 ± 0.10 vs. CG = 1.57 ± 0.21; *p* < 0.05	LDH (U·L) EG1 vs. CG ↓ EG2 vs. CG ↓ EG3 vs. CG ↓ EG4 vs. CG ↓ EG5 vs. CG ↔ CK (U·mL): EG1 vs. CG ↓ EG2 vs. CG ↓ EG3 vs. CG ↓ EG4 vs. CG ↓ EG5 vs. CG ↓
Zhu et al. [49]	To investigate the role of ME on muscle during exercise-induced fatigue both in vivo and in vitro	Mice: EG1 (*n* = 10) EG2 (*n* = 10) CG1 (*n* = 10) CG2 (*n* = 10)	EG: ME and caffeine CG: PL and PL + exercis	LDH	ME: EG1: 10 mL·kg^−1^ EG2: 10 mg·kg^−1^ caffeine CG1: 10 mL·kg^−1^ sterile water CG2: 10 mL·kg^−1^ sterile water + exercise	LDH (ng·L): EG1 = 25.46 ± 3.21 vs. CG2 = 33.85 ± 0.38; *p* < 0.05 EG2 = 24.78 ± 2.65 vs. CG2 = 33.85 ± 0.38; *p* < 0.05 CG2 = 33.85 ± 0.38 vs. CG1 = 27.35 ± 1.55; *p* < 0.05	LDH (ng·L): EG1 vs. CG2 ↓ EG2 vs. CG2 ↓ CG2 vs. CG1 ↑
Zhu et al. [29]	To explore the underlying mechanism of the MCP, a prescription for management of exercise-induced fatigue	Mice EG1 (*n* = 10) EG2 (*n* = 10) EG3 (*n* = 10) EG4 (*n* = 10) CG (*n* = 10	EG: MCP CG: PL	LDH	MCP EG1: 1.0 g·kg^−1^ MCP EG2: 2.0 g·kg^−1^ MCP EG3: 4.0 g·kg^−1^ MCP EG4: 10 mg·kg^−1^ caffeine CG1: 1.0 g·kg^−1^ sterile water CG2: 1.0 g·kg^−1^ sterile water + Ex	LDH (ng·L): EG1 = 28.0 ± 3.1 vs. CG1 = 30 ± 1; *p* > 0.05 EG1 = 28.0 ± 3.1 vs. CG2 = 34.2 ± 0.4; *p* < 0.01 EG2 = 24.9 ± 2.1 vs. CG1 = 30 ± 1; *p* > 0.05 EG2 = 24.9 ± 2.1 vs. CG2 = 34.2 ± 0.4; *p* < 0.01 EG3 = 28.2 ± 1.2 vs. CG1 = 30 ± 1; *p* > 0.05 EG3 = 28.2 ± 1.2 vs. CG2 = 34.2 ± 0.4; *p* < 0.01 EG4 = 23 ± 2.0 vs. CG1 = 30 ± 1; *p* > 0.05 EG4 = 23 ± 2.0 vs. CG2 = 34.2 ± 0.4; *p* < 0.01 CG1 = 30 ± 1 vs. CG2 = 34.2 ± 0.4; *p* < 0.05	LDH (ng·L): EG1 vs. CG1 ↔ EG1 vs. CG2 ↓ EG2 vs. CG2 ↔ EG2 vs. CG2 ↓ EG3 vs. CG1 ↔ EG3 vs. CG2 ↓ EG3 vs. CG1 ↓ EG4 vs. CG1 ↓ CG1 vs. CG2 ↓
**Blood markers of post-exertion protein degradation**							
**Authors**	**Objective**	**Participants or sample**	**Independent variable**	**Dependent variable**	**Supplementation protocol**	**Results**	**Effect**
Bilal et al. [66]	To investigate the effects of maca on serum indices and metabolic responses in racehorses	Racehorses: EG1 (*n* = 6) EG2 (*n* = 6) CG (*n* = 6)	EG: MPB CG: Basal diet	BUN	Maca root extract powder: EG1: Basal diet + 50 g·day EG2: Basal diet + 75 g·day CG: Basal diet	BUN (mg·dL): EG1 post-test = 11.50 vs. CG post-test = 12.00 EG12 post-test = 12.70 vs. CG post-test = 12.00	BUN (mg·dL): EG1 post test vs. CG ↓ EG2 post test vs. CG ↑
Chen et al. [54]	To investigate the effects of *L. meyenii* (maca) on hypoxia tolerance and fatigue relief and to determine its active constituents	Mice: EG1 (*n* = 12) EG2 (*n* = 12) EG3 (*n* = 12) EG4 (*n* = 12) EG5 (*n* = 12) EG6 (*n* = 12) EG7 (*n* = 12) EG8 (*n* = 12) EG9 (*n* = 12) CG (*n* = 12)	EG: MPB, ME and MWP CG: PL	BUN	MP: EG1 (high-dose): 1 g·kg^−1^ EG2 (medium-dose): 0.5 g·kg^−1^ EG3 (low-dose): 0.1 g·kg^−1^ MAE: EG4 (high-dose): 1 g·kg^−1^ EG5 (medium-dose): 0.5 g·kg^−1^ EG6 (low-dose): 0.1 g·kg^−1^ MWP: EG7 (high-dose): 1 g·kg^−1^ EG8 (medium-dose): 0.5 g·kg^−1^ EG9 (low-dose): 0.1 g·kg^−1^ CG: distilled water	BUN (mmol·L): MP groups: EG1 = 5.77 ± 0.63 vs. CG = 8.17 ± 0.55; *p* < 0.01 EG2 = 4.95 ± 0.03 vs. CG = 8.17 ± 0.55; *p* < 0.01 EG3 = 5.42 ± 0.23 vs. CG = 8.17 ± 0.55; *p* < 0.01 MAE groups: EG4 = 6.12 ± 0.09 vs. CG = 8.17 ± 0.55; *p* < 0.05 EG5 = 7.61 ± 0.56 vs. CG = 8.17 ± 0.55; *p* > 0.05 EG6 = 5.99 ± 0.36 vs. CG = 8.17 ± 0.55; *p* < 0.01 MWP groups: EG7 = 10.98 ± 1.81 vs. CG = 8.17 ± 0.55; *p* > 0.05 EG8 = 9.56 ± 1.05 vs. CG = 8.17 ± 0.55; *p* > 0.05 EG9 = 10.48 ± 1.62 vs. CG = 8.17 ± 0.55; *p* > 0.05	BUN (mmol·L): MP groups: EG1 vs. CG ↓ EG2 vs. CG ↓ EG3 vs. CG ↓ MAE groups: EG4 vs. CG ↓ EG5 vs. CG ↓ EG6 vs. CG ↓ MWP groups: EG7 vs. CG ↔ EG8 vs. CG ↔ EG9 vs. CG ↔
Li et al. [61]	To isolate and characterize the purified MP and specify the anti-fatigue composition of MP	Mice: EG1 (*n* = 10) EG2 (*n* = 10) EG3 (*n* = 10) EG4 (*n* = 10) CG (*n* = 10)	EG: MP CG: PL	BUN	MP-1 EG1: 100 mg·kg^−1^ EG2: 20 mg·kg^−1^ MP-2 EG3: 100 mg·kg^−1^ EG4: 20 mg·kg^−1^ CG: saline solution	BUN (mmol·L): EG1 = 8.97 ± 1.13 vs. CG = 14.35 ± 1.55; *p* < 0.05 EG2 = 11.2 ± 1.75 vs. CG = 14.35 ± 1.55; *p* < 0.05 EG3 = 8.35 ± 1.56 vs. CG = 14.35 ± 1.55; *p* < 0.05 EG4 = 11.25 ± 1.25 vs. CG = 14.35 ± 1.55; *p* < 0.05	BUN (mmol·L): EG1 vs. CG ↓ EG2 vs. CG ↓ EG3 vs. CG ↓ EG4 vs. CG ↓
Li et al. [63]	To investigate the anti-physical fatigue effect of MCP and the possible mechanisms	Mice: EG1 (*n* = 12) EG2 (*n* = 12) EG3 (*n* = 12) CG (*n* = 12)	EG: MCP CG: PL	BUN	MCP EG1: 500 mg·kg^−1^ EG2: 1000 mg·kg^−1^ EG3: 2000 mg·kg^−1^ CG: distilled water	BUN (mmol·L): EG1 = 10.45 ± 1.15 vs. CG = 10.7 ± 1.8; *p* > 0.05 EG2 = 9.99 ± 2.08 vs. CG =10.7 ± 1.8; *p* > 0.05 EG3 = 8.50 ± 1.50 vs. CG =10.7 ± 1.8; *p* < 0.05	BUN (mmol·L): EG1 vs. CG ↔ EG2 vs. CG ↔ EG3 vs. CG ↓
Li et al. [64]	To test the antifatigue effect of Xinjiang maca, to provide theoretical support for further development of health care products made of Xinjiang maca	Mice: EG1 (*n* = 40) EG2 (*n* = 40) EG3 (*n* = 40) CG (*n* = 40)	EG: Yellow maca root CG: PL	BUN	Maca treatment: EG1: 40 mg·kg^−1^ EG2: 400 mg·kg^−1^ EG3: 1200 mg·kg^−1^ CG: distilled water	BUN (mmol·L): EG1 = 18.50 ± 1.75 vs. CG = 11.15 ± 0.95; *p* < 0.05 EG2 = 15.25 ± 1.75 vs. CG = 11.15 ± 0.95; *p* < 0.05 EG3 = 30.25 ± 1.90 vs. CG = 11.15 ± 0.95; *p* < 0.05	BUN (mmol·L): EG1 vs. CG ↑ EG2 vs. CG ↑ EG3 vs. CG ↑
Li et al. [62]	To study the MP anti-fatigue activity for further development in industrial production.	Mice: EG1 (*n* = 10) EG2 (*n* = 10) EG3 (*n* = 10) CG (*n* = 10)	EG: MP CG: PL	BUN	MCP EG1: 150 mg·kg^−1^ EG2: 300 mg·kg^−1^ EG3: 600 mg·kg^−1^ CG: distilled water	BUN (nmol·L): EG1 = 8.6 ± 0.9 vs. CG = 10.4 ± 2.4; *p* < 0.01 EG2 = 9.45 ± 0.97 vs. CG = 10.4 ± 2.4; *p* > 0.05 EG3 = 9.65 ± 1.15 vs. CG = 10.4 ± 2.4; *p* > 0.05	BUN (nmol·L): EG1 vs. CG ↓ EG2 vs. CG ↔ EG3 vs. CG ↔
Liu et al. [65]	To investigate the anti-fatigue capacity of NBH	Mice EG1 (*n* = 6) EG2 (*n* = 6) CG (*n* = 6)	EG: ME and NBH CG: PL	BUN	ME EG1: 1000 mg·kg^−1^ extract of maca NBH EG2: 10 mg·kg^−1^ CG: distilled water	BUN (mmol·L) EG1 = 9.3 ± 0.6 vs. CG = 10.5 ± 0.3; *p* < 0.05 EG2 = 8.7 ± 0.5 vs. CG = 10.5 ± 0.3; *p* < 0.05	BUN (mmol·L) EG1 vs. CG ↓ EG2 vs. CG ↓
Tang et al. [50]	To investigate the antifatigue effect of MP was also evaluated by using a mouse weight-loaded swimming model to provide a theoretical basis and practical guidance for the comprehensive exploration of MP	Mice: EG1 (*n* = 20) EG2 (*n* = 20) EG3 (*n* = 20) CG (*n* = 20)	EG: MP CG: PL	BUN	MP EG1: 100 mg·kg^−1^ EG2: 50 mg·kg^−1^ EG3: 25 mg·kg^−1^ CG: distilled water	BUN (mmol·L): EG1 = 7.83 ± 0.52 vs. CG = 8.36 ± 1.12; *p* < 0.05 EG2 = 6.85 ± 0.98 vs. CG = 8.36 ± 1.12; *p* < 0.01 EG3 = 7.55 ± 0.53 vs. CG = 8.36 ± 1.12; *p* < 0.01	BUN (mmol·L): EG1 vs. CG ↓ EG2 vs. CG ↓ EG3 vs. CG ↓
Yang et al. [31]	To investigate the effects of macamides on endurance capacity and anti-fatigue properties in prolonged swimming mice	Mice: EG1 (*n* = 10) EG2 (*n* = 10) EG3 (*n* = 10) EG4 (*n* = 10) EG5 (*n* = 10) EG6 (*n* = 10) CG (*n* = 10)	EG: *N*-benzyllinoleamide, *N*-benzyloleamide and *N*-benzylpalmitamideCG: PL	BUN	*N*- benzyllinoleamide EG1: 12 mg·10 mL·kg^−1^ EG2: 40 mg·10 mL·kg^−1^ *N*-benzyloleamide EG3: 12 mg·10 mL·kg^−1^ EG4: 40 mg·10 mL·kg^−1^ *N*-benzylpalmitamide EG5: 12 mg·10 mL·kg^−1^ EG6: 40 mg·10 mL·kg^−1^ CG: distilled water	BUN (mmol·L): EG1 = 6.41 ± 0.66 vs. CG = 6.99 ± 0.76; *p* > 0.05 EG2 = 6.67 ± 0.83 vs. CG = 6.99 ± 0.76; *p* > 0.05 EG3 = 6.44 ± 0.99 vs. CG = 6.99 ± 0.76; *p* > 0.05 EG4 = 6.79 ± 1.00 vs. CG = 6.99 ± 0.76; *p* > 0.05 EG5 = 6.82 ± 0.84 vs. CG = 6.99 ± 0.76; *p* > 0.05 EG6 =7.13 ± 0.94 vs. CG = 6.99 ± 0.76; *p* > 0.05	BUN (mmol·L): EG1 vs. CG ↔ EG2 vs. CG ↔ EG3 vs. CG ↔ EG4 vs. CG ↔ EG5 vs. CG ↔ EG6 vs. CG ↔
Zheng et al. [60]	To investigate the effect of two macamides extracts on attenuating muscle damage	Mice: EG1 (*n* = 20) EG2 (*n* = 20) EG3 (*n* = 20) EG4 (*n* = 20) EG5 (*n* = 20) CG (*n* = 20)	EG: CME, PME, and Maca tablet CG: PL	BUN	CME EG1: 30 mg·kg^−1^ EG2: 120 mg·kg^−1^ PME EG3: 8 mg·kg^−1^ EG4: 32 mg·kg^−1^ Maca tablet EG5: 165 mg·kg^−1^ CG: aqueous solution	BUN (mmol·L) EG1 = 8.59 ± 0.23 vs. CG = 9.10 ± 0.38; *p* > 0.05 EG2 = 8.54 ± 0.25 vs. CG = 9.10 ± 0.38; *p* > 0.05 EG3 = 8.16 ± 0.22 vs. CG = 9.10 ± 0.38; *p* < 0.05 EG4 = 8.14 ± 0.23 vs. CG = 9.10 ± 0.38; *p* < 0.05 EG5 = 8.82 ± 0.27 vs. CG = 9.10 ± 0.38; *p* > 0.05	BUN (mmol·L) EG1 vs. CG ↔ EG2 vs. CG ↔ EG3 vs. CG ↓ EG4 vs. CG ↓ EG5 vs. CG ↔
Zhu et al. [49]	To investigate the role of ME on muscle during exercise-induced fatigue both in vivo and in vitro	Mice: EG1 (*n* = 10) EG2 (*n* = 10) CG1 (*n* = 10) CG2 (*n* = 10)	EG: ME and caffeine CG: PL and PL + exercise	BUN	ME: EG1: 10 mL·kg^−1^ EG2: 10 mg·kg^−1^ caffeine CG1: 10 mL·kg^−1^ sterile water CG2: 10 mL·kg^−1^ sterile water + exercise	BUN (μmol·L): EG1 = 106.3 ± 3.1 vs. CG1 = 84.5 ± 8.5; *p* < 0.05 EG1 = 106.3 ± 3.1 vs. CG2 = 119.4 ± 11.6; *p* < 0.05 EG2 = 99.4 ± 12.7 vs. CG2 = 119.4 ± 11.6; *p* < 0.05 CG2 = 119.4 ± 11.6 vs. CG1 = 84.5 ± 8.5; *p* < 0.05	BUN (μmol·L): EG1 vs. CG1 ↑ EG1 vs. CG2 ↓ EG2 vs. CG2 ↓ CG2 vs. CG1 ↑
Zhu et al. [29]	To explore the underlying mechanism of the MCP, a prescription for management of exercise-induced fatigue	Mice EG1 (*n* = 10) EG2 (*n* = 10) EG3 (*n* = 10) EG4 (*n* = 10) CG (*n* = 10	EG: MCP CG: PL	BUN	MCP EG1: 1.0 g·kg^−1^ MCP EG2: 2.0 g·kg^−1^ MCP EG3: 4.0 g·kg^−1^ MCP EG4: 10 mg·kg^−1^ caffeine CG1: 1.0 g·kg^−1^ sterile water CG2: 1.0 g·kg^−1^ sterile water + Ex	BUN (μmol·L): EG1 = 93 ± 1 vs. CG1 = 97 ± 3; *p* > 0.05 EG1 = 93 ± 1 vs. CG2 = 111 ± 6; *p* < 0.05 EG2 = 94 ± 9 vs. CG1 = 97 ± 3; *p* > 0.05 EG2 = 94 ± 9 vs. CG2 = 111 ± 6; *p* < 0.01 EG3 = 96 ± 6 vs. CG1 = 97 ± 3; *p* > 0.05 EG3 = 96 ± 6 vs. CG2 = 111 ± 6; *p* < 0.01 EG4 = 104 ± 3 vs. CG1 = 97 ± 3; *p* > 0.05 EG4 = 104 ± 3 vs. CG2 = 111 ± 6; *p* < 0.01 CG1 = 97 ± 3 vs. CG2 = 111 ± 6; *p* < 0.01	BUN (μmol·L): EG1 vs. CG1 ↔ EG1 vs. CG2 ↓ EG2 vs. CG1 ↔ EG2 vs. CG2 ↓ EG3 vs. CG1 ↔ EG3 vs. CG2 ↓ EG4 vs. CG1 ↔ EG4 vs. CG2 ↓ CG1 vs. CG2 ↓

BUN: blood urea nitrogen, CME: crude macamide extract, CG: control group, CK: creatine kinase, EG: experimental group, Ex: exercise, g: grams, g/kg: grams per kilogram, IU/L: international units per liter, LDH: lactate dehydrogenase, LME: liquid-soluble maca extract, ME: maca extract, mg/dL: milligrams per deciliter, mg/kg: milligrams per kilogram, mL/kg: milliliters per kilogram mmol/L: millimol per liter, MP: maca powder, MPB: maca powder blend, MCP: maca compound preparation, MWP: maca water polysaccharides, NBH: *N*-benzyl- 9z-12z-15z-octadecenamide, ng/L: nanograms per liter, nmol/L: nanomolar per liter, PL: placebo, PME: purified macamide extract, U/L: units per liter, U/mL: units per milliliter, μmol/L: micromol per liter, ↑: increase in the measured variable; ↔: no statistical changes in the evaluated variable; ↓: decrease in the measured variable.

**Table 2:** Meta-analysis summary by biomarker (overall) and by dose category, estimated with dependence-corrected models (three-level/robust variance estimation).

Biomarker	Dose	N Effects	N Studies	SMD [95% CI]	*p*-Value	*I*^2^ (%)	Model
CK	Low (all)	11	3	0.29 [−5.45, 6.03]	0.847	97.4	MLMA/RVE
LDH	All	39	10	−1.37 [−3.34, 0.59]	0.148	97.2	MLMA/RVE
LDH	Low	28	9	−1.21 [−3.82, 1.40]	0.317	98.0	RVE
LDH	Moderate	3	1	Not estimable (single study)	—	—	—
LDH	High	8	3	−1.24 [−11.62, 9.15]	0.652	98.3	RVE
BUN	All	43	11	−0.37 [−2.16, 1.42]	0.657	97.8	MLMA/RVE
BUN	Low	26	9	−0.68 [−2.67, 1.31]	0.455	97.3	RVE
BUN	Moderate	6	4	−0.68 [−5.44, 4.09]	0.641	98.4	RVE
BUN	High	11	5	1.24 [−6.04, 8.52]	0.660	99.2	RVE

SMD, standardized mean difference (Hedges’ g); CI, confidence interval; *I*^2^, proportion of total variability attributable to heterogeneity; MLMA, multilevel meta-analytic model; RVE, robust variance estimation (CR2 with Satterthwaite degrees of freedom). Estimates for CK and for dose subgroups with few clusters (e.g., the single-study LDH moderate-dose subgroup) are unstable and must be interpreted with caution.

**Table 3:** GRADE assessment of certainty of the evidence for each outcome.

Outcome	Risk of Bias	Inconsistency	Imprecision	Indirectness	Publication Bias	Certainty
CK	Not serious (CAMARADES adequate)	Serious (*I*^2^ = 97.4%)	Serious (very wide CI, non-significant)	Serious (animal models predominant)	Not reliably assessable (few studies)	Very low
LDH	Some concerns	Serious (*I*^2^ = 97.2%)	Serious (CI includes 0)	Serious (animal models predominant)	Not reliably assessable (few studies)	Very low
BUN	Not serious (CAMARADES adequate)	Serious (*I*^2^ = 97.8%)	Serious (CI includes 0)	Some indirectness (mixed models)	Not reliably assessable (few studies)	Very low

BUN: blood urea nitrogen, CI: confidence interval CK: creatine quinase, *I*^2^: proportion of total variability attributable to heterogeneity LDH: lactate dehydrogenase.

**Table 4:** Exploratory analyses of preparation type and dose as sources of heterogeneity, estimated with three-level/robust variance estimation models (LDH and BUN).

**Panel A. Subgroup Analysis by Preparation Type**				
Biomarker	Preparation	N Studies	SMD [95% CI]	*p*-value
LDH	Purified/isolated compound	7	−2.04 [−3.97, −0.12]	0.041
LDH	Whole/crude extract	4	−0.09 [−2.70, 2.52]	0.925
BUN	Purified/isolated compound	9	0.91 [−2.56, 4.39]	0.569
BUN	Whole/crude extract	3	−4.19 [−12.97, 4.58]	0.259
**Panel B. Meta-regression (preparation type + dose, ln mg/kg)**				
Biomarker	Moderator		β [95% CI]	*p*-value
LDH	Purified/isolated vs. whole/crude		−1.95 [−5.83, 1.93]	0.151
LDH	Dose (per 1-unit increase in ln mg/kg)		−0.08 [−0.67, 0.51]	0.737
LDH	Omnibus test of moderators (QM, *df* = 2)		—	0.319
BUN	Purified/isolated vs. whole/crude		5.38 [−34.74, 45.49]	0.352
BUN	Dose (per 1-unit increase in ln mg/kg)		0.42 [−0.10, 0.94]	0.094
BUN	Omnibus test of moderators (QM, *df* = 2)		—	0.381

SMD, standardized mean difference (Hedges’ g); CI, confidence interval; β, meta-regression coefficient. Analyses are exploratory and hypothesis-generating. CK was not modelled (no dose variation; three studies). Preparation type and dose are partially collinear (isolated compounds cluster at low mg/kg; whole/MCP preparations at high mg/kg), so their independent contributions cannot be fully separated.

## Data Availability

No new data were created or analyzed in this study.

## Abbreviations

The following abbreviations are used in this manuscript:

AST	aspartate aminotransferase
ATP	adenosine triphosphate
ATP-PC CYCLE	adenosine triphosphate-phosphocreatine cycle
CAMARADES	Collaborative Approach for the Analysis of Animal Data from Experimental Studies
CAT	catalase
CK	creatine kinase
CRP	C-reactive protein
EIMD	exercise-induced muscle damage
ES	effect size
GPx	glutathione peroxidase
GRADE	Grading of Recommendations Assessment, Development and Evaluation
CI	Confidence Interval
IL-6	interleukin-6
MDA	malondialdehyde
LDH	lactate dehydrogenase
MLMA	multilevel meta-analytic models
NAD	nicotinamide adenine dinucleotide
NADH	nicotinamide adenine dinucleotide + hydrogen
NOS	Newcastle Ottawa Scale
ROS	reactive oxygen species
RVE	robust variance estimation
SMD	standardized mean differences
SOD	superoxide dismutase
TNF-a	tumor necrosis factor-alpha
WOS	Web of Science
